# Apigenin-7-Glucuronide from *Urera aurantiaca* Inhibits Tumor Necrosis Factor Alpha and Total Nitrite Release in Lipopolysaccharide-Activated Macrophages

**DOI:** 10.1155/2020/6638764

**Published:** 2020-12-02

**Authors:** Carla Marrassini, Laura Cogoi, Valeria Sülsen, Claudia Anesini

**Affiliations:** Cátedra de Farmacognosia, Facultad de Farmacia y Bioquímica, Universidad de Buenos Aires, Consejo Nacional de Investigaciones Científicas y Técnicas, Instituto de la Química y Metabolismo del Fármaco (IQUIMEFA), Junín 956, C1113AAD Buenos Aires, Argentina

## Abstract

*Urera aurantiaca* is an Argentinean medicinal and edible species traditionally used to treat symptoms of inflammation. The aim of this study was to evaluate the anti-inflammatory activity of a methanol extract and its major compound. *U. aurantiaca* aerial parts were extracted with methanol by maceration. A phytochemical analysis was performed, and the extract's major component, apigenin-7-glucuronide (A7G), was identified by spectroscopic and HPLC methods. The analysis of the inflammatory mediators nitric oxide (NO) and tumor necrosis factor alpha (TNF-*α*) in lipopolysaccharide- (LPS-) stimulated macrophages were used in the evaluation of the extract and the major compound anti-inflammatory effects. The extract reduced LPS-augmented NO release from 100 *μ*g/mL (27%), reaching the highest inhibition at 1000 *μ*g/mL (96.3%), while A7G reduced it 30.7% at 1 *μ*g/mL, and its maximum effect was 97.1% at 10 *μ*g/mL. In the TNF-*α* model, the extract at 500 and 1000 *μ*g/mL reduced LPS-augmented TNF-*α* by 13.5% and 93.9%, respectively; meanwhile, A7G reduced it by 26.2% and 83.8% at 5 and 10 *μ*g/mL, respectively. *U. aurantiaca* popular use was validated. In the present study, for the first time, A7G was isolated from *U. aurantiaca*; furthermore, A7G showed anti-inflammatory effect in the macrophage cell line RAW264.7 (ATCC) and seems to be responsible for the extract anti-inflammatory effect.

## 1. Introduction

Human body's immune system reacts against injury generating inflammation. Injury can be caused by microbial invasions, antigen entrance, or other cell damage. Inflammation is part of a protective response searching for the restoration of the normal balance. In this complex process, endothelial cells, leucocytes, monocytes, and macrophages, soluble mediators, and reactive oxygen species (ROS) are involved. Although inflammation is a beneficial process, its duration is extremely important. Unrestrained inflammation leads to a sustained pathological status. Thus, chronic inflammation is part of various diseases in which asthma, atherosclerosis, cancers, chronic heart failure, diabetes mellitus, metabolic syndrome-associated disorders, and osteoporosis, among others, are included [1].

Macrophages have an important role in the generation and development of the inflammation process through the secretion of cytokines in response to a stimulus. Taking advantage of this particular behavior, in this study, a RAW264.7 (ATCC) macrophage cell line was used for the evaluation of potential anti-inflammatory agents in two inflammation models.

*Urera aurantiaca* Wedd. (Urticaceae) is an Argentinean native plant that can also be found in Bolivia, Brazil, Paraguay, and Uruguay and is known with the common names *pica pica*, *pino guasú*, *ortiga, ortiga colorada*, *urtiga*-*branca,* and *urtiguinha*. This herb is popularly used in the treatment of rheumatic and tooth pain, for inflammation symptoms, in case of varicose veins, furuncles, bruises, dermal diseases, and trauma [2]. Its roots have been used for the preparation of diuretic teas [3–5]. It is also used for hair loss, abdominal pain, and hemorrhoids [6]. Its leaves and fruits are edible, and the leaves' content in protein and minerals such as calcium, magnesium, iron, and sulfur is remarkable [6, 7].

In previous works, *U. aurantiaca in vivo* anti-inflammatory and antinociceptive effects [8], its modulatory effect on immune and tumoral cells during inflammation [9], and its *in vitro* anti-inflammatory and antioxidant activities [10] have been reported. However, *U. aurantiaca* chemical composition is unknown.

The aim of this study was to identify the main component of the *U. aurantiaca* methanolic extract and evaluate the anti-inflammatory effect of the extract and the compound in LPS-activated macrophages.

## 2. Materials and Methods

### 2.1. Plant Material

Dr. R. H. Fortunato collected and identified *Urera aurantiaca* Wedd. (Urticaceae) in March 2008 in Paraguay: Cerro Corá National Park, Naranjahai (22°38′S; 58°36′W, 288 m elevation, 2.5 m altitude), Amambay Department. A voucher specimen, number 9339, was deposited at Pharmacobotanical's Museum, Pharmacy and Biochemistry Faculty, Buenos Aires University, Argentina.

### 2.2. Preparation of the Plant Extract

*Urera aurantiaca* aerial parts were dried and ground into a fine powder. It was extracted by maceration with solvents in the crescent polarity order: methylene chloride, ethyl acetate, and methanol. The methanol extract (UAP) was lyophilized. Its yield was 1.81%.

### 2.3. Isolation and Identification of the Extract's Major Compound

The extract's major compound was isolated by preparative paper chromatography performed on Whatman no. 3 using n-BuOH : AcOH : H_2_O (4 : 1 : 1) as solvents. A7G was identified by UV and EIMS and confirmed by HPLC in comparison with an external standard retention times and UV spectra in three different HPLC systems, taking into account literature data.

### 2.4. High-Performance Liquid Chromatography

Three HPLC methods were performed in order to identify the extract's major compound. A Varian 9000 instrument equipped with a diode array detector and a Rheodyne injector fitted with a 20 *μ*l loop was used. A C18 column (Agilent 5 *μ*m, 150 mm × 4.6 mm) was employed for the following three methods:

  Method 1: mobile phase A: H_2_O/AcOH (98 : 2) and B: MeOH/AcOH (98 : 2). Elution gradient: 15–40% B, 30 min; 40–75% B, 10 min; 75–85% B, 5 min. Flow rate: 1.2 mL/min. Detection: 325 nm [11].
  Method 2: mobile phase A: H_2_O/formic acid (98 : 2) and B: MeOH/formic acid (98 : 2). Elution gradient: 15–40% B, 15 min; 40–75% B, 5 min; 75–85% B, 5 min; 85–100% B, 5 min; 100% B, 5 min. Flow rate: 0.8 mL/min. Detection: 325 nm.
  Method 3: mobile phase A: 0.5% phosphoric acid in water and solvent B: 0.5% phosphoric acid in acetonitrile. Elution gradient: 8% B, 1 min; 8–25% B, 19 min; 25% B, 7 min; 25–100% B, 3 min; 100–8% B, 2 min; 8% B, 10 min. Flow rate: 1.2 mL/min. Detection: 330 nm.

### 2.5. Macrophage Culture Conditions

The American Type Culture Collection (ATCC) RAW264.7 murine macrophage cell line was cultured in a colorless Dulbecco's Modified Eagle's medium which was supplemented with 10% FBS, 2 mM glutamine, 100 IU/mL penicillin, and 100 *μ*g/mL streptomycin. Cells were kept in a humidified incubator with 5% CO_2_ at 37°C.

### 2.6. Total Nitrite Determination

Total nitrite was measured as an indicator of NO production. Macrophages were incubated for 24 h in medium (basal conditions) or with LPS (1 *μ*g/mL) alone or in the presence of different concentrations of the extract or A7G. After incubation, all supernatants were collected to determine the NO levels and the cells were used to determine proliferation. The nitrite accumulated in the culture medium was determined by the Griess reaction [12]: 100 *μ*L of the supernatant was mixed with 100 *μ*L of Griess reagent (50 *μ*L of 1% sulfanilamide solution in 5% phosphoric acid and 50 *μ*L of 0.1% naphthylene diamine dihydrochloride solution). The absorbance was read at 540 nm after 20 min incubation. Serial dilutions of a NaNO_2_ solution were used to build a standard curve.

### 2.7. Cell Proliferation

Cell proliferation was determined by the reduction in 3-(4,5-dimethylthiazol-2-yl)-2,5-diphenyltetrazolium bromide (MTT). After 24 h incubation and separation of the supernatant, the cells were incubated for 4 h in 100 *μ*L medium and 10 *μ*l MTT (5 mg/mL). The formazan formed was dissolved in acidified isopropanol (0.04 N HCl in isopropanol). The absorbance was read at 540 nm.

### 2.8. TNF-*α* Determination

TNF-*α* was determined in the supernatant of cultured macrophages using the commercial Cayman Chemical kit TNF-*α* (mouse) ELISA kit in accordance with the manufacturer's recommendations. Macrophages were incubated for 24 h in medium (basal conditions) or with LPS (1 *μ*g/mL) alone or in the presence of different concentrations of the extract or A7G. After incubation, all supernatants were collected to determine the TNF-*α* level.

### 2.9. Statistical Analysis

Data were expressed as the average of triplicate values of three independent experiments. Multiple comparisons were performed by analysis of variance (ANOVA) and Dunnett's test. *p* < 0.05 was considered statistically significant.

## 3. Results and Discussion

Taking into account *U. aurantiaca* popular use, the aim of this study was to evaluate a methanol extract anti-inflammatory activity and identify its active compounds. For this purpose, a phytochemical analysis was performed. Two *in vitro* inflammation models were used to assess the sample activity.

**Figure 1:**
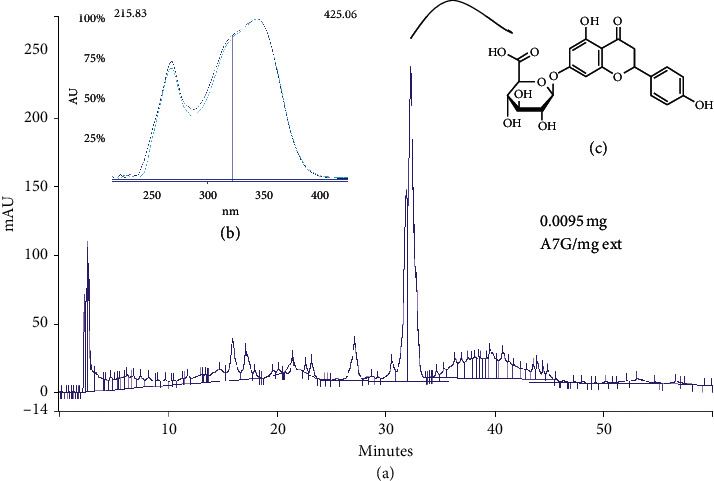
A: Extract HPLC profile in system 1: major peak retention time (Rt): 31.12 min (A7G standard, data not shown, Rt: 31.15 min). B: UAP major peak and A7G standard superposed UV spectra. C: A7G molecular structure.

First, the extract was analyzed and fractioned in order to isolate and identify its major compound. A7G (0.0095 mg/mg extract) was obtained as a light-yellow powder. UV (*λ*_max_, nm): MeOH: 268, 325; NaOMe: 272, 339, 391; AlCl_3_: 277 (sh), 300 (sh), 323 (sh), 358; AlCl_3_ + HCl: 277 (sh), 315, 358; NaOAc: 268, 283 (sh), 335 (sh), 393; NaOAc + HBO_3_: 269, 335 (sh), 397. EIMS (70 eV) *m*/*z* (% rel. int.): 269 [M-Glu]+ (2.6), 176 [Glu-H]+ (73.1). This is the first time A7G is reported in *U. aurantiaca*. UV spectra and HPLC retention times of the major peak and a standard were compared in three different systems. A cochromatography was also performed (data not shown). In Figure 1, the extract HPLC profile obtained when method 1 was performed is shown.

A7G is derived from the flavones apigenin that is a nontoxic and nonmutagenic flavonoid, present in fruits and vegetables such as cardoon, celery, artichoke, parsley, and others, some of which are widely marketed as dietary and herbal supplements [13]. It is known that A7G presents anti-inflammatory effect and is currently prescribed to treat inflammatory diseases such as upper respiratory infections [14, 15]. This is the first time A7G is described in *U. aurantiaca*.

LPS can be used as an external substance to generate macrophage activation and consequent release of inflammatory mediators [16]. In response to an infectious challenge, bacterial components such as LPS induce monocyte differentiation into classically activated macrophages, which, once activated, are able to kill pathogens through phagocytosis, produce ROS, NO, enzymes, and inflammatory cytokines such as TNF-*α* [17]. The proinflammatory cytokine TNF-*α* and the reactive free radical NO are inflammatory mediators secreted by macrophages. Macrophages are involved not only in the inflammation progress but also in the development of inflammatory diseases. In this study, TNF-*α* and NO were evaluated using macrophages incubated with LPS in presence of different concentrations of the extract or A7G.

**Figure 2:**
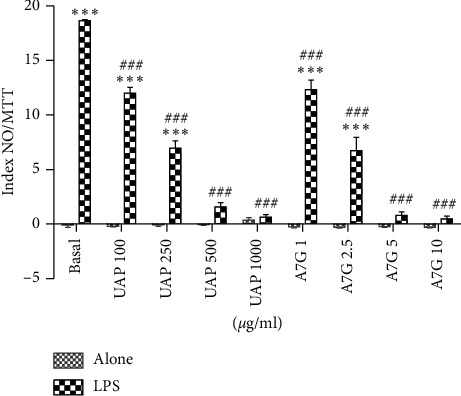
Effect of A7G and the extract (UAP) on nitrite release. The results are expressed as index NO/MTT and represent means (SEM of three experiments made in triplicate). Basal: cells without treatment; LPS: cells previously treated with LPS. The statistical differences were determined by ANOVA followed by Dunnett's test (^*∗∗∗*^*p* < 0.001, statistically different respect to basal; ^###^*p* < 0.001, statistically different respect to LPS).

As expected, the treatment of RAW264.7 cells with LPS increased NO levels (Figure 2). At basal conditions, neither the extract nor the compound modified NO level. Under LPS effect, the extract decreased NO production in all the doses tested, in a concentration-dependent manner. The extract was able to reduce NO-augmented release by 27% from the lowest dose, 100 *μ*g/mL. The maximum inhibitory effect was 96.3% at 1000 *μ*g/mL. Moreover, the extract caused a complete reversion of NO level, reaching basal levels at 500 and 1000 *μ*g/mL, in agreement with what had been observed before [10]. A7G also showed an anti-inflammatory effect from the lowest dose, 1 *μ*g/mL, representing 30.7% inhibition, and in a concentration-dependent manner. Furthermore, its maximum inhibitory effect was 97.1% inhibition (10 *μ*g/mL). A7G was also able to revert LPS effect (5 and 10 *μ*g/mL). It is interesting to note that the A7G tested concentrations are in the order in which A7G is present in the extract, so the effect observed for the extract could be related principally to A7G.

**Figure 3:**
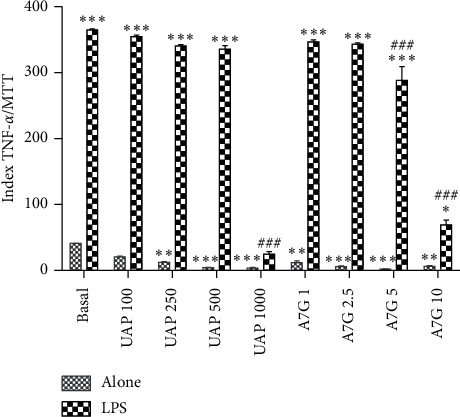
Effect of A7G and the extract (UAP) on TNF-*α* concentration. The results are expressed as index TNF-*α*/MTT and represent means (SEM of three experiments made in triplicate). Basal: cells without treatment; LPS: cells previously treated with LPS. The statistical differences were determined by ANOVA followed by Dunnett's test (^*∗*^*p* < 0.05, ^*∗∗*^*p* < 0.01, and ^*∗∗∗*^*p* < 0.001: statistically different respect to basal; ^#^*p* < 0.05, ^##^*p* < 0.01, and ^###^*p* < 0.001: statistically different respect to LPS).

The extract and A7G effect was also evaluated in the TNF-*α* assay (Figure 3). Neither the extract nor the major compound activity on TNF-*α* had been described before. TNF-*α* is a major proinflammatory cytokine which regulates inflammation-related disorders by inducing different pathways and signaling molecules such as NF-*ĸ*B. NF-*ĸ*B is a transcription factor that regulates gene expression in the setting of the inflammatory process. NF-*ĸ*B plays an essential beneficial role in normal physiology, but its inappropriate regulation is implicated in the pathogenesis of several diseases. At basal conditions, the extract and the compound were capable of decreasing TNF-*α* level. Also, A7G and the extract significantly decreased TNF-*α* level in a concentration-dependent manner in LPS-stimulated macrophages. The maximum inhibition effect was 93.9% for the extract (1000 *μ*g/mL) and 97.1% for A7G (10 *μ*g/mL). These were also the doses in which the LPS effect was completely reverted, reaching basal levels. A7G seems to be responsible for the effect exerted by the extract.

## 4. Conclusion

In this work, A7G, *U. aurantiaca* major compound, was identified for the first time. A7G and *U. aurantiaca* methanol extract activity upon inflammatory mediators such as NO and TNF-*α* were evaluated. Both the flavonoid and the extract prevented the inflammatory reaction induced by LPS in RAW264.7 cells, decreasing TNF-*α* and nitrite release. *Urera aurantiaca* popular use as an anti-inflammatory agent was consequently validated. A7G seems to be responsible for the extract anti-inflammatory effect.

## Data Availability

The data used to support the findings of this study are included within the article.

## Abbreviations

A7G: Apinenin-7-glucuronide
LPS: Lipopolysaccharide
MTT: 3-(4,5-dimethylthiazol-2-yl)-2,5-diphenyltetrazolium bromide
NF-*ĸ*B: Nuclear factor *ĸ*B
NO: Nitric oxide
ROS: Reactive oxygen species
TNF-*α*: Tumor necrosis factor alpha
UAP: *Urera aurantiaca* methanol extract.

## Supplementary Materials

Supplementary Materials Graphical summary of the *in vitro* anti-inflammatory activity of *Urera aurantiaca* methanol extract and its main compound, apigenin-7-glucuronide: the inflammatory mediators nitric oxide (NO) and tumor necrosis factor-alpha (TNF-*α*) in lipopolysaccharide- (LPS-) stimulated macrophages were used in the evaluation of the extract and the major compound anti-inflammatory effects. Graphs in green show total nitrite levels and TNF-*α* level of untreated cells and LPS- or LPS + extract-treated cells. Graphs in violet show the inflammatory mediator responses obtained for apigenin-7-glucuronide-treated cells.
