# Supplementary material for: Apigenin-7-Glucuronide from Urera aurantiaca Inhibits Tumor Necrosis Factor Alpha and Total Nitrite Release in Lipopolysaccharide-Activated Macrophages
Source: Evid Based Complement Alternat Med. 2020 Dec 2;2020:6638764. doi: 10.1155/2020/6638764 (PMC7725547; doi:10.1155/2020/6638764)
Supplement: Supplementary Materials — Graphical summary of the in vitro anti-inflammatory activity of Urera aurantiaca methanol extract and its main compound, apigenin-7-glucuronide: the inflammatory mediators nitric oxide (NO) and tumor necrosis factor-alpha (TNF-α) in lipopolysaccharide- (LPS-) stimulated macrophages were used in the evaluation of the extract and the major compound anti-inflammatory effects. Graphs in green show total nitrite levels and TNF-α level of untreated cells and LPS- or LPS + extract-treated cells. Graphs in violet show the inflammatory mediator responses obtained for apigenin-7-glucuronide-treated cells. [file 6638764.f1.docx]

**Graphical abstract:**
